# Melange

**Published:** 1885-09

**Authors:** 


### MELANGE.

THE FIRST FRUITS.—Dr. W. A. Morris' paper on "Incision of the Sphincter Vaginæ," which appeared in our July number, is attracting considerable attention. A singular coincident occurred in connection with it. Dr. R. H. L. Bibb, of Saltillo, Mexico, formerly of Austin, writes to Dr. Morris that he was called in consultation in a wealthy Mexican family, to a case of labor in a primipara. He had just received that number of

DANIEL'S MEDICAL JOURNAL, and took it with him; and, during his ride, read the paper referred to. Arriving, he found a case urgently requiring episiotomy, to prevent laceration; and without a moment's hesitation, he incised the sphincter. The child was expelled with considerable force, and all went well; no laceration of the perineum resulting.

---

THE DENGUE is prevailing in Austin; most of the Doctors have had it, and are now sympathizing with their patients.

---

AN OFF-SET.—The declination of the famous 28 was calculated to make an impression that they represented the sentiment of the profession of Pennsylvania. This has been effectively negatived by the signature of some four hundred Pennsylvania physicians to a document endorsing the action of the Association, and sustaining the code. The list embraces fourteen Ex-Presidents of the State Medical Association, and amongst the whole are the names of such men as Pancoast, Hewson, Lawrence Trumbull, Atkinson, Dunglison,Sutton, Leffmann,Formad,etc., all quite as "eminent" as any of the light brigade of decliners.

---

GALVESTON is to have waterworks.

---

A WISE PRECAUTION.—Mr. Samson: "I really must get my life insured, I am not feeling very well. I think I will call on Dr. Gilbert and get some medicine, and to-morrow I will get insured." Mrs. Samson: "Don't you think, Tommy dear, it would be safer to get insured first?"—*Puck.*

---

THE NEW STATE HOUSE.—We learn from the *Sanitary News*, of Chicago, that the contract for the iron, for this building seven million pounds, and to cost $300,000, has been awarded to a firm in Antwerp, through their Boston agent, and that there is any amount of "virtuous indignation aroused among the iron manufacturers of America" in consequence. The foreigners simply outbid them, it being, the contractor says, a question of dollars and cents. The Pulman Car-wheel Works, of Chicago, furnished the iron for the first floor and basement columns, now in place. The Diebold Lock Company will furnish the vault work. J. C. McFarland the galvanized iron work and slating, and Eaton & Prince the elevators. Carnegie Bros. & Co., of Pittsburg, got the contract for the iron for the railroad from Burnet to the granite quarries. These contracts, given out in Chicago last week, aggregate $1,000,000. The railroad to the quarries is to be built at once, and, as soon as it is finished, work on the Capitol will be resumed. It is to be completed by January 1, 1891—four years and four months from the present time.

---

Inoculation against Yellow Fever Successful.—Domingoes Friere writes from Rio Janiero to the Editor of the *Sanitary News*, Chicago, that the fever did not assume the proportion of former years because of his successful inoculation of all classes, ages and nationalities with his "attenuated culture." He says he inoculated four thousand, not one of whom had the fever. and that it was often done in the houses where deaths had just occurred. He says, "This preventive measure has succeeded in a marvelous way, the disease being extinguished in its home."

---

Has anybody any recollection of a so-called International Congress having been held in America in 1876? It seems to have gone out of the memory of the oldest inhabitant.

---

A Suggestion for the Columbus (O.) Medical Journal.—

Ding Dong Dire; the Fat's in the Fire!
What did the trick? The little Texas Kick.

---

He Hit It.—In filing out a "birth certificate," in a case where the father's name was a little doubtful, a Chicago physician, with a readiness equal to any emergency, promptly filed the blank with "E Pluribus Unum."—*Exchange.*

---

Death of Two Young Ladies by a Druggist's Mistake.—Mr. Am. Ende, Hoboken's popular druggist, a man of learning and skill, made a fatal mistake on a prescription which the Doctor brought himself. The Doctor was engaged to a young lady—Miss Gretchen—the daughter of a well-to-do man in New York, and visiting her, found her and her sister ill. He prescribed quinine, and brought the medicine himself, and administered it with his own hand. Shortly afterwards, the youngest lady was found in a comatose state, and soon died. Miss Gretchen soon followed, despite efforts of several physicians. Mr. Ende called, and, taking up the powders, was horrified to find he had put up morphine instead of quinine as ordered. His anguish of mind is said to have been pitiable. He went to his house and attempted to take his own life by atropia, but the physicians saved him, and he is under arrest. The father of the young ladies sent messages of sympathy to Mr. Ende, and has had prescriptions filled at his shop since, says the *N. Y. Medical Journal.* Much sympathy is felt for the bereaved parents and, also, for the unfortunate druggist, whose mind is in a state bordering on madness. The law of New York makes it criminal, with a penalty, if, by ignorance or carelessness of the druggist in dispensing medicine, a person loses his life. This was not ignorance, but it certainly was carelessness. It is to be hoped that in this case, justice will be largely tempered with mercy. Mr. Ende was so popular with the physicians, on account of his skill and learning, as well as for his reputation for being a consciencious and pains-taking prescriptionist, that although his place is in Hoboken, they bring and send him prescriptions from New York and even Brooklyn. He says he cannot account for the mistake except that his attention was distracted by persons in the shop, who persisted in conversing with him while he was busy. The frequent occurrence of mistaking morphine for quinine, it would seem, would suggest the necessity of having morphine colored some strong color which should be characteristic of that drug alone. This item of news appears in the New York Journals of the 5th.

---

THE VERY LATEST.—As we go to press we are distressed to learn, and to announce, that Dr. Jones, of Jonesville, has "declined." That settles it; the Congress has gone up.

---

A GOOD PUN AND A NEAT COMPLIMENT.—Dr. Thos. F. Wood, editor of that excellent Journal (*N. C. Medical Journal*), in an editorial, makes a comparison between the British Medical Association and the American Medical Association, and between the Journals of the two great societies, he says: "The editor has all the qualities of an earnest heart." For fear that somebody may fail to see the point, we will venture to give them a hint by saying that Mr. Ernest Hart, editor *British Journal*, is said to be ambitious to go to Parliament. Dr. Wood would hardly attribute the "qualities" of a politician to our Dr. Davis.

---

POISON IN ICE CREAM.—In San Antonio, Texas, recently, quite a number of persons were poisoned by eating ice cream flavored with vanilla extract. It is not known how it occurred. The symptoms were those of strychnine and narcotic combined, with a little tartarized antimony thrown in, to-wit: convulsions, opisthotonos, frontal pains, stupor, together with violent emesis and catharsis. All recovered.

---

QUARANTINE APPOINTMENTS.—Dr. John McKnight has been appointed, by the State Health Officer, Sanitary Inspector at Laredo; and Dr. McKinney, at El Paso. State Health Officer Swearingen visited those gates of Mexican commerce and travel the latter part of August, to give instruction to the Quarantine officers, and to inspect the Stations.

---

THE OX AND THE FLY.—There is one very important point in connection with this declining business that these gentlemen seem not to have duly considered. It is this: they are not of so much consequence in the estimation of others, as they are in their own, and that of their special (and specialist) friends. Their assumption that there can be no Congress without *them*, and that the whole of Europe will stay away on their account, reminds one of the story of the fly on the ox's horn—"If I am too heavy Mr. Ox," etc.

---

NIPPED IN THE BUD.—Had we less regard for veracity, we might exclaim, in the first flush of disappointment, "'Twere ever thus from childhood's hour, I've seen my fondest hopes decay," etc. Were we to so exclaim now, we would be guilty of that for which Ananias is said to have suffered; for amongst our "fondest hopes"—indeed, we may say, "chief amongst ten thousand" fond hopes, and "altogether lovely"—was the desire to make this journal a success. In our pardonable pride and gratification, we point to the complimentary and cordial greeting extended us by our colleagues, (see last page in this book), as evidence of success, so far; and, although a little irrelevant just now, we tender our most grateful acknowledgments. Thus our hopes, instead of decaying, have been stimulated. This aroused our ambition to be the first to give a report of the now famous Committee's meeting held in N. Y., on the 3rd. We held our pages open for the report. We had a friend present who was to send it: we thought we would strike it just right. He wrote; but "phancy our pheelings," when we read, "Dear Doctor:—I hasten to write that you may not keep your Journal waiting. *I was pledged to secrecy.* No reports will be furnished for publication until the proceedings appear in the Association Journal." Well, now, that is paralizing. If the Association organ has a corner on all such, we will quit.

